# FGF21 drives a shift in adipokine tone to restore metabolic health

**DOI:** 10.18632/aging.100565

**Published:** 2013-06-13

**Authors:** Andrew Charles Adams, Alexei Kharitonenkov

**Affiliations:** Lilly Research Laboratories, Indianapolis, IN 46285 USA

Fibroblast growth factor 21 (FGF21) is a key metabolic regulator with significant potential to treat metabolic disease. Rather than traditional glucose or lipid centric therapies, FGF21 instead functions via expansive remodeling of whole body energy balance [1]. While FGF21's ability to ameliorate disease burden is well established in species ranging from mice to men, the mechanism(s) by which FGF21 is able to instigate the plethora of its in vivo actions have yet to be elucidated. Recently, adipose tissue has emerged as a critical target organ where FGF21 engages its primary receptor complex (FGFR1/KLB) [2, 3]. In fat, FGF21 acts as a metabolic rheostat serving to modulate secretion of adipokines which in turn mediate discrete aspects of FGF21-induced physiology. Supportive of this proposed mode of action is recent evidence demonstrating that adiponectin is not only a biomarker for FGF21 target engagement but is also essential for facilitating some of FGF21s downstream actions [4, 5]. Of critical importance, acute FGF21 signaling is not compromised in Adn−/− mice when compared to their WT counterparts. However, only in WT animals with intact adiponectin signaling is FGF21 able to trigger a cascade of metabolic events leading to correction of hyperglycemia and hyperinsulinemia. Correlated with the lack of improvement in glycemia and insulin sensitivity there was also lack of ceramide lowering in FGF21 treated Adn−/− animals. Supportive of partitioning of FGF21s metabolic endpoints, weight loss, elevated energy expenditure and reduced circulating lipids were yet evident FGF21-treated Adn−/− mice.

Of note, induction of adiponectin is also a hallmark of another class of anti-diabetic therapeutics, the thiazoladinediones [6]. Indeed, it was recently suggested that FGF21 may play a role in mediating the physiological consequences of TZD treatment [7]. In concordance with this hypothesis, upon administration of either FGF21 or PPARγ ligands elevation of serum adiponectin reaches similar levels in mice. Nevertheless, TZDs mainly impact transcription of adiponectin gene while FGF21 functions as a potent adiponectin secretagogue [4, 5]. Furthermore, it has also recently been reported that in vivo the FGF21 & PPARγ pathways do not in fact intersect [8]. These distinctions are important in reconciling the lack of rosiglitazone-associated side effects such as adipose accrual and elevation of liver enzymes in FGF21 treated mice [4, 8]. Further work delineating additional downstream mediators of FGF21 action in adipose and other tissues will be critical to understanding of its mechanism of action and thus potentially yielding novel therapeutic avenues.

**Figure 1:**
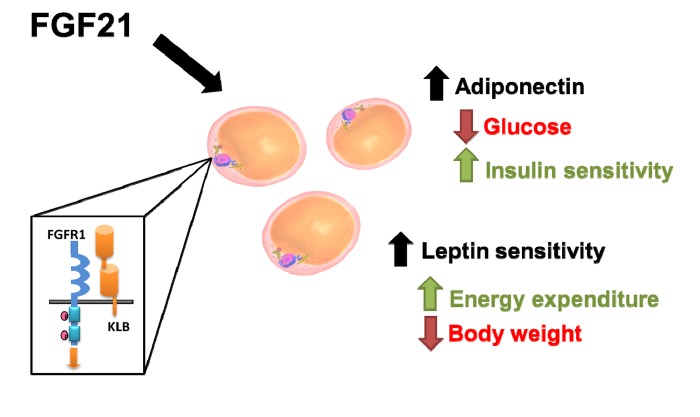
Effects of chronic FGF21 treatment in vivo are mediated by specific hormonal pathways downstream of adipose tissue activation.
